# Supplementary material for: Chromothripsis during telomere crisis is independent of NHEJ, and consistent with a replicative origin
Source: Genome Res. 2019 May;29(5):737–49. doi: 10.1101/gr.240705.118 (PMC6499312; doi:10.1101/gr.240705.118)
Supplement: Supplemental Material [file supp_gr.240705.118_Supplemental_file_1.zip › contigs/annotated_contigs/DB93/contig.2.DB93_length_325_mean_cov_23.1353846154.docx]

**DB93_length_325_mean_cov_23.1353846154**

```
AGAGGGACATTGGGACATATCTCTGCACTGATCGCTGAGGTGATGTAAAACTTGTCTAGGCTTTGCCTACAGTGGGATTTATGA|CATA
 >chrUn_gl000216:159150-159264 + E=3e-51 p=0e+00                                     >ch
TTTCTGCACTGATCACCCAGGTGATG|GACTCTTGTCTTGGATCTGCCTATGGGGGCATAGTGACATATCTCTGCACTGATCACTCATG
r18:109908-110123 + E=4e-97
TGATGTAACGCTTGTCTAAGCTGCGCCTAAAGGGGAATTGTGACAGATATCTCCACTGATCACCCAGGTGATGTAACTCTTGTCTAGGC

TCTGCTTACAGGGGGTATTGTGAGATATCTCTGC|TTTGGTCCCCTAAGTGATGTAACAAT
```
